# Implications of TDP-43 in non-neuronal systems

**DOI:** 10.1186/s12964-023-01336-5

**Published:** 2023-11-23

**Authors:** Hao Ke, Kang Liu, Baowei Jiao, Limin Zhao

**Affiliations:** 1 Human Aging Research Institute (HARI) and School of Life Science, Nanchang University, and Jiangxi Key Laboratory of Human Aging, Nanchang, 330031 China; 2 Ganzhou People’s Hospital, Ganzhou, 341000 China; 3 National Key Laboratory of Genetic Evolution & Animal Models, Kunming Institute of Zoology, Chinese Academy of Sciences, Kunming, Yunnan 650201 China; 4 KIZ-CUHK Joint Laboratory of Bioresources and Molecular Research in Common Diseases, Kunming Institute of Zoology, Chinese Academy of Sciences, Kunming, 650201 China

**Keywords:** Embryonic development, Fat metabolism, Mammary gland development, Spermatogenesis, Stem cell, TDP-43, Tumor, Viral infection

## Abstract

TAR DNA-binding protein 43 (TDP-43) is a versatile RNA/DNA-binding protein with multifaceted processes. While TDP-43 has been extensively studied in the context of degenerative diseases, recent evidence has also highlighted its crucial involvement in diverse life processes beyond neurodegeneration. Here, we mainly reviewed the function of TDP-43 in non-neurodegenerative physiological and pathological processes, including spermatogenesis, embryonic development, mammary gland development, tumor formation, and viral infection, highlighting its importance as a key regulatory factor for the maintenance of normal functions throughout life. TDP-43 exhibits diverse and sometimes opposite functionality across different cell types through various mechanisms, and its roles can shift at distinct stages within the same biological system. Consequently, TDP-43 operates in both a context-dependent and a stage-specific manner in response to a variety of internal and external stimuli.

Video Abstract

**Supplementary Information:**

The online version contains supplementary material available at 10.1186/s12964-023-01336-5.

## Introduction

Encoded by the *TARDBP* gene, TDP-43 is highly conserved across various species [1]. The protein was initially recognized as a transcription repressor (with a size of 43 kD, hence its name), exhibiting affinity for the TAR DNA sequence of human immunodeficiency virus type 1 (HIV-1) [2]. Subsequent study identified TDP-43 as a constituent of ubiquitinated insoluble aggregates found in the brains of individuals with frontotemporal lobar dementia (FTLD) [3], with more recent research establishing strong associations between TDP-43 and a range of neurodegenerative disorders.

TDP-43 is a multifunctional protein that operates within a precise regulatory framework. Heterozygous knockout of TDP-43 in mice does not result in successful TDP-43 knockdown due to its own negative feedback regulation, while homozygous knockout of TDP-43 leads to death in both embryonic and adult stages [4–6]. This precise regulation highlights the importance of TDP-43 in fundamental life processes. In addition to its established functions in neurodegenerative diseases, TDP-43 is reported to participate in diverse developmental stages [4, 7–9]. Therefore, in this review, we discuss the important functions of TDP-43 in different tissues and developmental stages in non-neurodegenerative systems. Notably, current research suggests that TDP-43 plays a context-dependent and stage-specific role in different environments and developmental stages. Furthermore, TDP-43 likely serves as an important stress sensor, responding to intracellular and extracellular stimuli to maintain homeostasis in life processes and activities.

## Structure and function of TDP-43

**Fig. 1:**
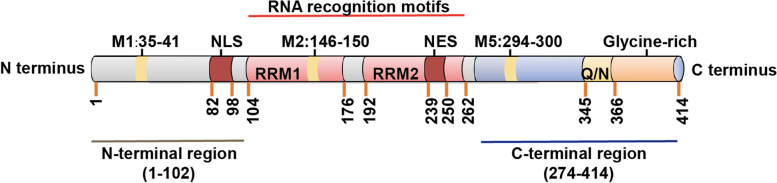
Structure of TDP-43 protein. Numbers represent amino acid lengths of TDP-43. NLS: nuclear localization signal, RRM: RNA recognition motifs, NES: nuclear export signal, Q/N: glutamine/asparagine-rich domain, M1–M5: five putative mitochondrial localization signals

As part of the heterogeneous nuclear ribonucleoprotein (hnRNP) family, TDP-43 is primarily located in the nucleus, although it can also exist within the cytoplasm and mitochondria [10–12]. The structural composition of TDP-43 includes an N-terminal region with a nuclear localization signal (NLS), two RNA recognition motifs (RRM1 and RRM2), a nuclear export signal (NES) located within RRM2, a C-terminal region featuring a glutamine/asparagine-rich (Q/N) domain and a glycine rich region, as well as five putative mitochondrial localization signals (M1–M5) (Fig. 1) [13–17]. The N terminal domain is thermodynamically stable and well-folded and undergoes reversible oligomerization [18, 19]. Several reports have proposed that N-terminal domain-induced dimerization of TDP-43 is necessary for its physiological functions, such as RNA splicing [16, 20]. While the NLS and NES are both implicated in the nucleocytoplasmic shuttling of TDP-43, with the NLS additionally facilitating the nuclear transport of TDP-43 [19, 21], the precise functionalities of the NES remain controversial [15].

**Fig. 2:**
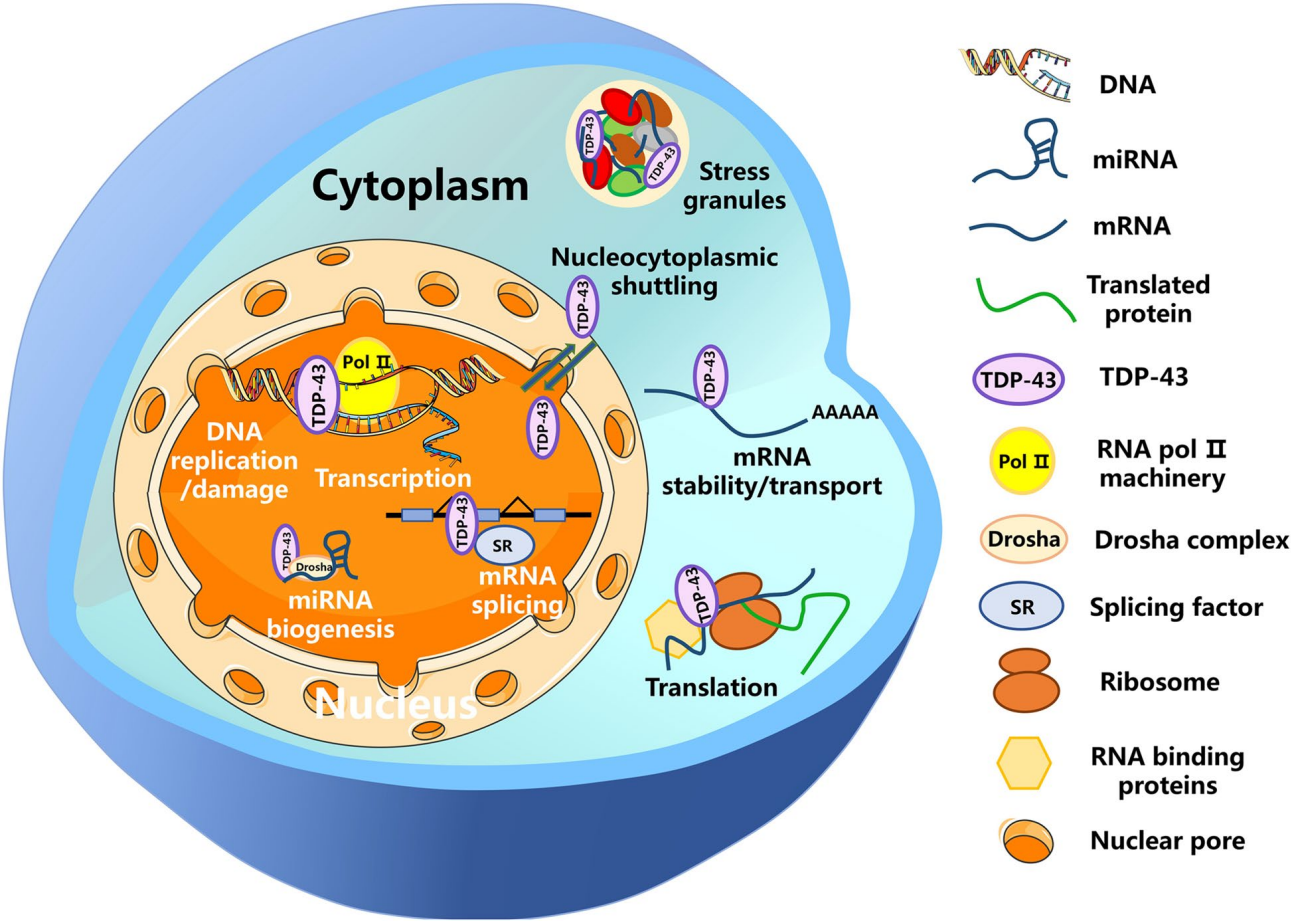
Functions of TDP-43 protein. TDP-43 functions as a nucleocytoplasmic shuttling protein via nuclear pores, which can regulate cellular functions at multiple levels. At the DNA level, TDP-43 is involved in gene transcription, DNA damage, and DNA replication. At the RNA level, TDP-43 is involved in RNA splicing, RNA transport, and RNA stability. TDP-43 is also involved in miRNA biogenesis, stress granules, and various other cellular processes

TDP-43 participates in many functions (Fig. 2) and can act as a DNA-binding protein. TDP-43 was initially studied as a transcriptional inhibitor that binds to the TAR regulatory element of HIV-1, thereby influencing transcription factor assembly [2]. Subsequent investigations revealed that TDP-43 binds to the TGTGTG domains in the promoter region of the mouse *SP-10* gene, leading to the inhibition of gene transcription and impacting sperm formation [7]. Additional studies have also demonstrated that TDP-43 is involved in DNA damage, DNA replication, and genome stability [22–24].

As a splicing factor, TDP-43 can affect alternative splicing of various genes, such as apolipoprotein A-II [25], *RXRG* [26], *SC35* [27], *SMN* [28], *ETF1* [26], *BRCA1* [26], schizophrenia-associated TNIK gene [29], *PAR3*/*NUMB* [30], and cancer stem cell marker *CD44* [31]. TDP-43 also exerts influence on various other RNA processes, including RNA transport and stability [32], RNA translation [33], and microRNA (miRNA) biogenesis [34]. In conjunction with fragile X syndrome protein (FMRP), TDP-43 can cooperatively suppress the translation initiation of *Map1b*, *Rac1*, and *GluR1* mRNAs [33]. In addition, TDP-43 forms associations with 126 proteins to jointly regulate mRNA transport and stability in HEK-293 cells [35], and its overexpression can induce profound RNA destabilization [36]. Interestingly, TDP-43 appears to play a dual role in RNA stability, both promoting mRNA instability, as observed for CDK6 mRNA decay [37, 38] and tau mRNA instability [39], and maintaining mRNA stability, as observed for *G3BP1* [40], *Add2* [41], *RPTOR/RAPTOR* [42], *Btn1a1*, and *Xdh* [8]. Moreover, TDP-43 not only regulates targeted mRNA stability, but also stabilizes mitochondrial transfer RNA (mt-tRNA) in human mitochondria [43]. Thus, these studies collectively demonstrate the involvement of TDP-43 in various RNA metabolic processes.

## TDP-43 in degenerative neurological diseases

TDP-43 is a well-recognized neurodegenerative disease-related protein. In 2006, ubiquitinated and hyperphosphorylated TDP-43 was identified in both amyotrophic lateral sclerosis (ALS) and FTLD [3, 44]. Subsequently, abnormal aggregation of TDP-43 has been implicated in various other neurodegenerative diseases. These pathological inclusions of TDP-43, known collectively as “TDP-43 proteinopathy”, are characterized by the accumulation of hyperphosphorylated, ubiquitinated, and cleaved TDP-43 in the cytoplasm, with a simultaneous reduction of TDP-43 levels in the nucleus. Nevertheless, the precise mechanisms underlying TDP-43 proteinopathy in neurodegenerative diseases remain to be fully elucidated.

**Fig. 3:**
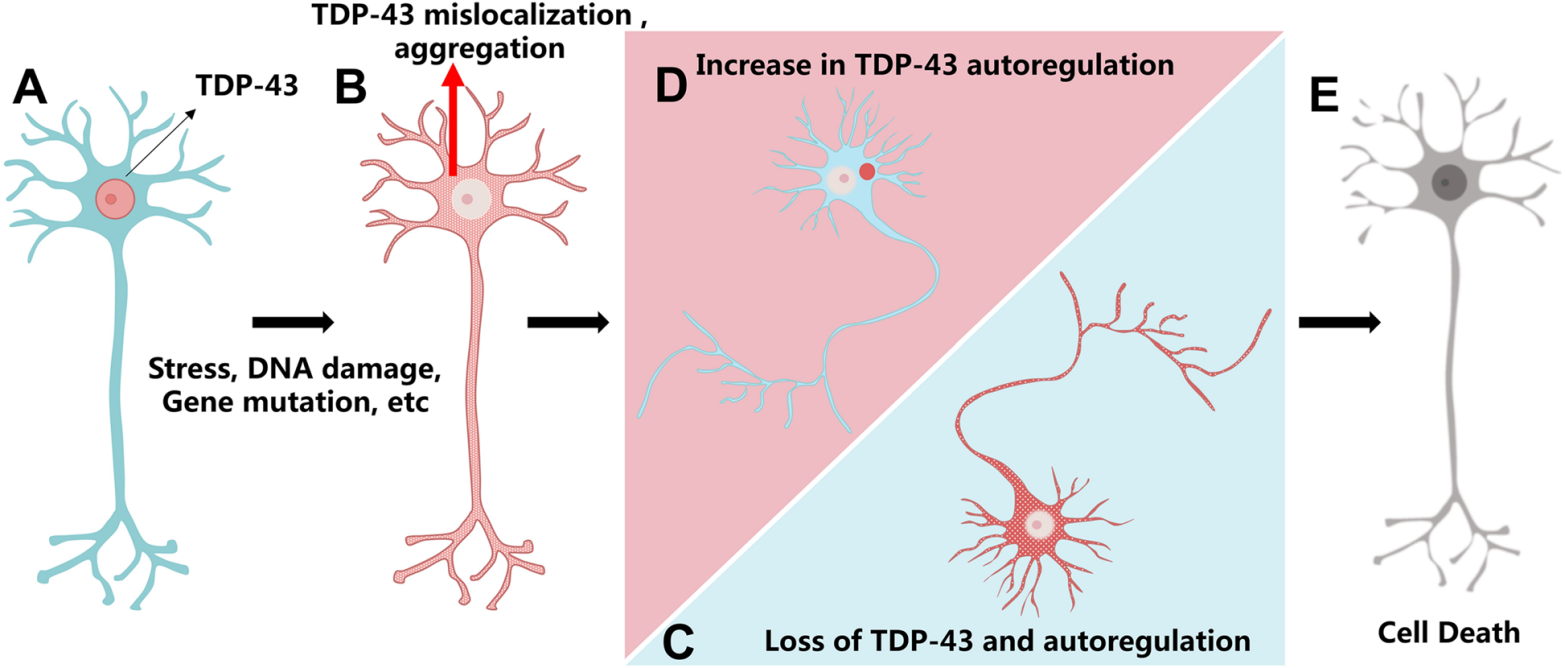
Models of TDP-43 toxicity. **A** Under normal conditions, TDP-43 expression is under strict regulation and is predominantly located in the nucleus. **B** When subjected to stress, such as DNA damage, the TDP-43 protein moves from the nucleus to cytoplasm, where it forms phosphorylated pre-inclusion bodies. **C** This leads to the loss of normal nuclear TDP-43. If TDP-43 autoregulation occurs within the nucleus, loss of TDP-43 stimulates the cell to produce more TDP-43 protein, thereby aggravating the accumulation of TDP-43 in the cytoplasm. **D** If TDP-43 autoregulation occurs within the cytoplasm, the increase in cytoplasmic TDP-43 can result in an increase in TDP-43 autoregulation and a decrease in the synthesis of new TDP-43 protein. **E** Regardless of whether TDP-43 autoregulation occurs in the nucleus or cytoplasm, it ultimately leads to neuronal death

At present, various theories exist regarding the cytotoxicity caused by the mislocalization of TDP-43 from the nucleus to the cytoplasm (Fig. 3). The first theory postulates that neuronal exposure to specific stressors triggers the cytoplasmic mislocalization of TDP-43, leading to the formation of phosphorylated pre-inclusion bodies within the cytoplasm, thereby sequestering free TDP-43 protein and depleting normal nuclear TDP-43 (Fig. 3A, B). In response, the nucleus initiates a compensatory mechanism to generate more TDP-43 protein to restore its normal nuclear function (Fig. 3C). However, the increased production of TDP-43 exacerbates its accumulation in the cytoplasm, ultimately resulting in cell death (Fig. 3E). An alternative hypothesis posits that the formation of pre-inclusion bodies by TDP-43 in the cytoplasm triggers an automatic regulation response in the cytoplasm, leading to a reduction in TDP-43 protein synthesis (Fig. 3D). Given the essential role of TDP-43 in neuronal cells, the down-regulation of its protein synthesis can also lead to normal nuclear TDP-43 deprivation and subsequent neuronal death [45, 46] (Fig. 3E).

## TDP-43 in non-neurodegenerative diseases

### TDP-43 in cancer

The involvement of TDP-43 in cancer was identified as early as two decades ago, with notable associations found between common variants near *TARDBP* and *EGR2* and the incidence of Ewing sarcoma [47]. In more recent years, research has expanded our understanding of the role of TDP-43 in the progression of different cancers, including breast cancer, cervical cancer, lung cancer, hepatocellular carcinoma, glioblastoma, and melanoma.

#### TDP-43 acts as an oncogenic factor to promote tumor progression

In the context of breast cancer, multiple reports, including our own, have suggested that TDP-43 may act as an oncogenic factor to promote tumor progression. Notably, Fang et al. found that curcumin, a dietary pigment with known anticancer activities, can significantly inhibit TDP-43 expression in the breast cancer cell line MCF7 [48]. In our previous study, we observed elevated levels of TDP-43 in triple-negative breast cancer (TNBC), which showed a significant correlation with poor prognosis. Furthermore, we found TDP-43 knockdown led to a significant reduction in tumor progression, including proliferation and metastasis, accompanied by extensive changes in splicing events [9]. In subsequent studies, we demonstrated that TDP-43 can directly bind to the pre-mRNA of CD44, thereby regulating its alternative splicing [31]. Given that CD44 is a well-known marker in breast cancer stem cells (BCSCs), the influence of TDP-43 on CD44 alternative splicing may accelerate cancer progression.

In relation to lung cancer, TDP-43 has been found to enhance the metastasis of non-small cell lung cancer cells (NSCLC) through its regulation of *MALAT1* expression [49]. TDP-43 also promotes the growth and metastasis of NSCLC as a downstream effector of the long noncoding RNA (lncRNA) *MIAT* [50]. In advanced NSCLC patients carrying sensitized epidermal growth factor receptor (EGFR) mutations, the use of EGFR-tyrosine kinase inhibitors (TKIs) in combination with targeted therapy drugs is considered a viable treatment option, but can result in the eventual development of EGFR-TKI resistance in patients. Interestingly, the lncRNA *LCETRL3*, which is located at 4q12 and is associated with resistance to EGFR-TKI [51], can partially diminish the effectiveness of EGFR-TKIs by stabilizing TDP-43 [52], implying potential involvement of TDP-43 in the development of resistance to EGFR-TKIs.

Park et al. found that TDP-43 can regulate glycolysis by modulating the phosphofructokinase isoform through miRNA 520, with TDP-43 knockdown leading to impaired glucose metabolism and cell proliferation in multiple HCC cell lines [53]. Guo et al. also found that TDP-43 can induce epithelial-mesenchymal transition and promote HCC metastasis by stimulating the Wnt/β-catenin signaling pathway [54]. Liu et al. further reported that TDP-43 can suppress apoptosis in HCC by up-regulating the lipid metabolism modulator ABHD2 [55].

TDP-43 also plays an important role in various other cancers. Similar to its effects in HCC, TDP-43 promotes the proliferation and migration of melanoma cells, potentially through modulation of glucose metabolism [56]. The TDP-43-HDAC6 signaling axis in glioblastoma multiforme (GBM) acts as a stress-responsive pathway, driving GBM progression and activating autophagy to promote cell survival under nutrient deprivation [57]. Furthermore, using the human bone osteosarcoma (U2OS) cell line, Ayala et al. revealed that loss of TDP-43 can affect nuclear membrane stability and increases apoptosis [37].

#### TDP-43 plays dual roles in tumor progression

In addition to promoting cancer progression, TDP-43 can also function as a tumor suppressor. For example, Kim et al. found that a high TDP-43 expression is necessary during TRIM16-induced cancer cell death, with both TRIM16 and TDP-43 serving as good prognostic markers in neuroblastoma and breast cancer [58]. Zaman et al. reported the higher *TARDBP* microarray values are associated with a significant increase in overall survival in pediatric neuroblastoma [59]. Lee et al. provided evidence that elevated levels of TDP-43 in HeLa cells can induce G2/M arrest and cell death via a p53-dependent mechanism [60].

TDP-43 also affects diverse functions of downstream genes. In HeLa cells, TDP-43 inhibits CDK6 by recruiting UG-rich *Cdk6* transcripts [37], whereas in CHO-K1 cells, it activates CDK6 expression ^[38]^. Additionally, TDP-43 exhibits a dual role in the complex regulation of cancer-related miRNAs, acting as a promoter of cancer progression through the regulation of miR-423-3p, while also exerting inhibitory effects on cancer progression through the regulation of miR-500a-3p [61].

These studies underscore the heterogeneity of cancer and highlight the intricate regulatory role of TDP-43 in cancer progression. Although the mechanisms by which TDP-43 regulates cancer are complex and not yet fully understood, they likely depend on the specific cellular context and cancer type.

#### Potential correlation of TDP-43 between cancer and neurodegenerative diseases

Accumulating evidence suggests the existence of an inverse comorbidity phenomenon between oncological and neurodegenerative conditions. In 2014, Ibanez et al. conducted a transcriptomic meta-analysis of several neurodegenerative diseases and three cancers (lung, prostate, and colorectal cancer). They discovered a significant overlap between up-regulated genes in neurodegenerative diseases and down-regulated genes in cancer, as well as down-regulated genes in neurodegenerative diseases and up-regulated genes in cancer [62]. Subsequent studies have revealed that many genes, proteins, and signaling pathways regulated in both cancer and neurodegenerative diseases display contrasting patterns. Notably, the well-known tumor suppressor p53 is up-regulated in AD, PD, and Huntington’s disease (HD), but down-regulated in many cancers [63–65]. Given the multifaceted nature of TDP-43, it is plausible to consider the interconnected roles it plays in both cancer and neurodegenerative diseases.

Furthermore, the intricate mechanisms underlying the effects of TDP-43 in neurodegenerative diseases can be elucidated from various perspectives, including point mutations within the genome, multiple splicing mutants, and phosphorylation proteins. However, it remains to be investigated whether TDP-43 mutations and truncations exist and hold significant roles in cancer. Additionally, given its role as a splicing factor, the phosphorylation status of TDP-43 directly impacts its splicing function, prompting inquiry into the presence of abnormal phosphorylation of TDP-43 in tumor cells. While exogenous overexpression of TDP-43 can lead to elevated expression of the 35 kD isoform, which may exert cytotoxic effects on cancer cells and induce cell death [66], further in-depth studies are necessary to comprehensively understand the precise function of TDP-43 in tumorigenesis.

### TDP-43 in development

TDP-43 is expressed in almost all cell types and is highly conserved among different species. Increasing evidence confirms its crucial regulatory role in various developmental processes, including embryonic development, body fat metabolism, mammary gland development, the reproductive system and so on.

#### TDP-43 in embryonic development

Various studies utilizing knockout mice have provided compelling evidence for the crucial roles of TDP-43 in embryonic development. Researchers have previously constructed TDP-43 knockout mice by targeting the translation initiation site of exon 2 of *Tardbp* mRNA [4, 67], with such models demonstrating that homozygous deletion of TDP-43 can lead to peri-implantation lethality. Notably, despite morphological normality *in vitro*, blastocysts with homozygous *Tardbp* deletion exhibit impaired outgrowth in the inner cell mass. Conversely, mice with heterozygous TDP-43 deletion display normal development and fertility, with no noticeable abnormalities up to 14 months of age and no changes in TDP-43 protein expression compared to wild-type mice [4]. Sephton et al. [5] also reported that while homozygous *Tardbp* knockout mice died between ED 3.5 and 8.5, heterozygous *Tardbp* mice showed no differences in TDP-43 expression or phenotypic changes, including body weight, growth rate, appearance, and fertility, and further displayed no gross tissue abnormalities up to the age of 6 months compared to control littermates [5]. In another study, Chiang et al. inserted loxp sites on both sides of the third exon of *Tardbp* mRNA and produced a nonfunctional truncated TDP-43 variant in mice after crossing with the CAG-Cre transgenic mouse line [6, 68]. In accordance with the above studies, their mouse model yielded fertile heterozygous knockout mice with normal TDP-43 expression but failed to produce viable homozygous TDP-43 knockout mice due to embryonic death at around day 7.5 [6].

Collectively, these different mouse models demonstrate that homozygous loss of TDP-43 can result in embryo death during blastocyst implantation, confirming the necessity of TDP-43 expression in embryonic development. In addition, protein levels in heterozygous *Tardbp* knockout mice remained stable across these models, indicating that TDP-43 possesses an inherent regulatory mechanism to mitigate the adverse effects of TDP-43 deletion.

Regarding the mechanism by which TDP-43 safeguards embryonic development, a recent study conducted in mouse embryonic stem cells (mESCs) discovered that TDP-43 protects the embryonic genome by interacting with L1 open reading frame 1 protein (L1 ORF1p) [46].

#### TDP-43 in fat metabolism

In addition to its vital role in embryonic development, the potential functions of TDP-43 in adult animals have been increasingly investigated. To overcome the issue of embryonic death caused by TDP-43 deletion, researchers have employed conditional knockout mice to study the physiological function of TDP-43. For example, Chiang et al. crossed floxed *Tardbp* mice with *Rosa26-ErCre* mice (which express Cre protein upon tamoxifen induction) to generate inducible *Tardbp* knockout mice. Upon tamoxifen induction, these conditional homozygous *Tardbp* knockout mice unexpectedly died by day 9, characterized by substantial fat loss and increased fatty acid consumption. Mechanistically, deletion of TDP-43 down-regulated the expression of obesity-associated gene *Tbc1d1*, leading to alterations in body fat metabolism [6].

Accumulating evidence supports the involvement of TDP-43 in fat metabolism. Stallings et al. demonstrated that TDP-43 regulates fat homeostasis and glucose metabolism [69]. Coughlan et al. showed that high-fat jelly diets in TDP-43^A315^^T^ mutant mice (ALS model) can restore bioenergetic balance and extend lifespan [70]. Egawa et al. revealed that TDP-43 can reduce the expression of sterol regulatory element-binding protein 2 (SREBP2), and thus regulate cholesterol biosynthesis [71]. Li et al. demonstrated that TDP-43, acting as a transcription suppressor, alleviates the inhibition of the downstream target gene *Cyp8b1* by binding with the lncRNA lncLSTR, thus regulating systemic lipid metabolism in mice [72]. Studies have also identified the involvement of TDP-43 in obesity pathogenesis [73]. In our previous work, we revealed TDP-43 as a key regulator of milk fat metabolism. Notably, through targeted TDP-43 knockout in the mammary gland from middle pregnancy to the lactation stage, we found that TDP-43 loss leads to abnormal milk fat metabolism, resulting in lactation failure and death of infant mice due to inadequate nutrition [8].

Lipids serve as essential components of biological membranes, not only providing efficient energy storage for organisms, but also acting as signaling molecules/messengers in diverse developmental pathways [74], with the regulation of fat metabolism by TDP-43 further highlighting its importance during development.

#### TDP-43 in the reproductive system

Precise regulation of cell type-specific gene transcription is crucial for spermatogenesis, where a multitude of testis-related genes are activated in a programmed spatiotemporal order. For example, *Acrv1*, which codes for the sperm acrosomal protein SP-10, is strictly expressed at the transcriptional level in the testes and round spermatids. Evidence has shown that the proximal promoter of *Acrv1* is sufficient to maintain round spermatid-specific expression and acts as an insulator, preventing ectopic expression of *Acrv1* in somatic cells [75]. TDP-43 was first defined as a putative regulator that binds to the *Acrv1* promoter via two GTGTGT-motifs, eventually inhibiting premature *Acrv1* expression during spermatogenesis [76]. TDP-43 was subsequently identified as a transcriptional repressor in spermatocytes, suppressing *Acrv1* gene transcription in a histone deacetylase-independent manner [77]. In somatic cells, TDP-43 can act as an insulator protein to prevent *Acrv1* ectopic expression [78]. These findings demonstrate that TDP-43 can maintain spermatogenesis through precise and diverse regulatory mechanisms.

Subsequent investigations have revealed that TDP-43 is expressed in both Sertoli cells and germ cells, indicating its potential adoption of multiple conformational states during different stages of spermatogenesis. Studies have shown that TDP-43 expression initiates in type B/intermediate spermatogonia, reaches its peak in preleptotene spermatocytes, disappears in leptotene and zygotene spermatocytes, reappears in pachytene spermatocytes and early round spermatids, and subsequently decreases in later spermatids [79]. The varied expression and localization patterns of TDP-43 suggest that it plays a multifaceted role in spermatogenesis. Indeed, deficiency in TDP-43 is linked with impaired spermatogenesis and male infertility, as observed in the germ cells of fertile and subfertile men [80]. Further studies involving TDP-43 knockout in Sertoli cells and male germ cells have confirmed its crucial role in the male reproductive system [81, 82]. Campbell et al. demonstrated that TDP-43 loss in mouse spermatogonia can initiate meiotic failure, resulting in fewer and more morphologically abnormal sperm accompanied by severely reduced fertility [82]. Zomer et al. also observed that TDP-43 loss in mouse Sertoli cells can induce spermatogenesis failure and male subfertility [81]. These findings collectively establish the importance of TDP-43 during spermatogenesis, suggesting that TDP-43 could potentially serve as an essential indicator of male factor infertility.

#### TDP-43 in mammary gland development

Lactation, a highly distinctive feature of mammals, provides offspring with sufficient nutrition to ensure their survival. In earlier research, we found that TDP-43 is required for mammary gland development [8, 83].

In our previous studies focused on identifying key post-transcriptional regulatory genes during lactation and exploring the impact of positive selection on species divergence, we conducted an evolutionary analysis of RNA-binding proteins (RBPs) across 15 mammalian species. Our analysis revealed that TDP-43 underwent positive selection within the mammalian lineage, after applying false discovery rate correction. Subsequently, we investigated the expression patterns of TDP-43 during different stages of mammary gland development in mice and observed high expression levels during pregnancy and early lactation. Notably, TDP-43 knockout in the mouse mammary gland resulted in a significant reduction in milk production, with subsequent starvation of the pups within three weeks postpartum. Furthermore, we collected human milk from lactating women and analyzed the expression level of TDP-43 in isolated RNA from the milk lipids. Remarkably, we found a positive correlation between TDP-43 expression and higher milk output, suggesting that TDP-43 mRNA expression may serve as a potential indicator of lactation in parturient women [8]. Furthermore, we observed that conditional knockout of TDP-43 in the pubertal mammary gland had a significant inhibitory effect on mammary epithelial proliferation and mammary gland repopulation [83]. This finding is particularly significant as pubertal mammary gland development serves as the basis for subsequent breast lactation. Thus, these results provide evidence that TDP-43 may promote milk secretion at multiple levels.

#### TDP-43 in stem cells and other developmental processes

Stem cells are necessary for tissue development, homeostasis, and repair [84]. Abnormal differentiation of stem cells can have profound consequences on the development of individuals and organs [85, 86]. Multiple studies suggest that TDP-43 participates in the regulation of stem cell development. For example, Modic et al. showed that TDP-43 regulates pluripotency-differentiation transition in ESCs by regulating *Sox2* alternative polyadenylation. Additionally, TDP-43 is also reported to regulate the expression of the *Neat1* gene, repressing the formation of paraspeckles. Notably, during differentiation, TDP-43 is recruited to paraspeckles and facilitates the exit of ESCs from pluripotency and embryonic patterning [87]. Moreover, our previous study further revealed the importance of TDP-43 as a critical regulator in mammary stem cells [83].

TDP-43 is also implicated in various other developmental processes. Regarding myogenesis, Militello et al. found that TDP-43 binds to the muscle-enriched lncRNA *Myolinc* and regulates muscle-related gene expression, while knockdown of TDP-43 inhibits myogenic differentiation [88]. Furthermore, Xia et al. verified that CHCHD10 interacts with TDP-43 to promote myofiber regeneration and newly differentiated myotubes during myogenesis [89]. Li et al. revealed that H19 may act as a scaffold to recruit TDP-43 to the promoter region of myogenic differentiation (MYOD) in porcine satellite cells (PSCs), thereby activating the transcription of MYOD, and leading to PSC differentiation [90]. In the context of islets, TDP-43 is associated with early-stage insulin secretion through CaV1.2-induced exocytosis [91]. TDP-43 is also reported to regulate mammalian spinogenesis through translational repression of Rac1 [92].

### TDP-43 in viral infection

Initially recognized as a protein associated with HIV, TDP-43 has been increasing implicated in the entry, replication, and latency of other viruses [93] beyond HIV [2, 94, 95], including hepatitis B virus (HBV) [96], enteroviruses (EVs) [97–99], herpes simplex virus-2 (HSV) [93, 100], Theiler’s murine encephalomyelitis virus (TMEV) [101], human endogenous retrovirus K (HERV-K) [102, 103], West Nile virus (WNV) [93, 104], and severe acute respiratory syndrome coronavirus 2 (SARS-CoV-2) [105–107]. However, the function of TDP-43 appears to vary markedly among individuals, depending on the specific viral context.

Multiple studies have provided evidence that TDP-43 acts as a protective factor in individuals during viral infections. Ou et al. first cloned and characterized TDP-43 in 1995, demonstrating its binding to the TAR sequence motifs of HIV-1 and inhibition of *HIV-1* gene expression [2]. Although subsequent reports indicated that TDP-43 expression in human immune cells is unrelated to HIV-1 replication [108], more recent observations have indicated that TDP-43 affects HIV viral envelope glycoprotein complex (Env) fusion, infection capacities, and viral production [94, 95]. Additionally, TDP-43 is also involved in the antiviral innate immune response. Notably, upon viral infection, TDP-43 is released from the lncRNA *Malat1* and undergoes cleavage to form the TDP-35 isoform. This process promotes the production of IRF3-initiated antiviral type I interferons (IFNs) by preventing IRF3 proteasomal degradation, thereby enhancing host defense against viral infection [90].

TDP-43 may also have negative effects on individuals. For example, during HBV infection, TDP-43 acts as a host factor that stimulates gene transcription by binding to the HBV core promoter, forms complexes with other proteins supporting the HBV life cycle, and inhibits pregenomic HBV RNA splicing, thereby promoting the production of HBV replicative intermediates, mRNAs, proteins, and virions [96].

## Conclusions and perspectives

### TDP-43 in mammalian life cycle

**Fig. 4:**
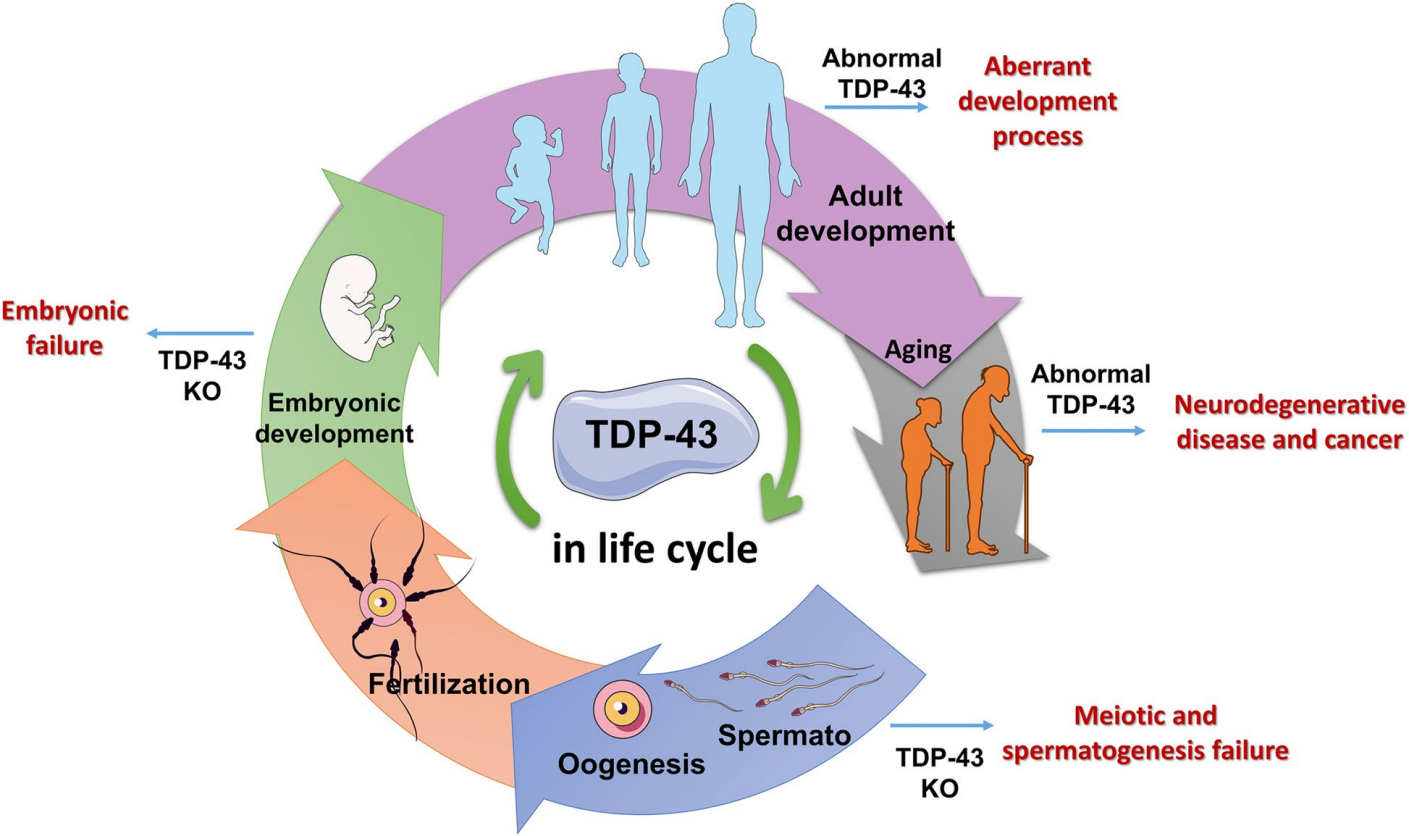
Functions of TDP-43 in human life cycle. TDP-43 is reported to participate in spermatogenesis, embryonic development, adult development, and aging. Abnormal TDP-43 expression and location can lead to developmental failure and disease

The above findings highlight the wide-ranging roles of TDP-43 throughout various stages of mammalian life, encompassing early development to aging (Fig. 4).

Mammalian early embryonic development commences with the fusion of egg and sperm, giving rise to a totipotent zygote that develops into a fully formed individual. The production of sperm and eggs depends on germ cell meiosis. TDP-43 has been established as an essential protein for the completion of prophase I during spermatogenesis, and its deletion can lead to meiotic arrest, spermatogenesis failure, and low fertility in male mice [82] (Fig. 4A). However, as meiosis is not exclusive to spermatogenesis, also occurring during oogenesis, it would be worth exploring whether the absence of TDP-43 also impacts oogenesis and female fertility. In addition, as meiosis is not unique to mammals and TDP-43 exhibits a high degree of conservation across different species, it is likely that TDP-43 is essential for the reproductive system in all sexually reproducing animals. Although the specific involvement of TDP-43 in the fertilization process and zygote formation remains uncertain (Fig. 4B), it is evident that TDP-43 is crucial for embryonic development (Fig. 4C). Various gene knockout models have confirmed that TDP-43 loss results in the death of pre-implantation embryos, emphasizing the significance of TDP-43 as a key regulatory gene in both sperm and egg formation and subsequent embryonic development [4–6, 46].

TDP-43 is involved in many life processes during adult development after birth (Fig. 4D, E). Firstly, systemic deletion of TDP-43 in postnatal mice causes rapid body fat loss and subsequent death, suggesting that TDP-43 is necessary for adult survival [6]. Secondly, TDP-43 is a key regulatory factor in mammary gland development and milk secretion, playing a crucial role in lactation and newborn survival [8, 83]. In addition, tissue-specific knockout or *in vitro* reduction of TDP-43 expression in other organs has confirmed that TDP-43 is also associated with stem cells [83, 87], muscle generation [88, 89], neural development [109–111], insulin secretion [91], and systemic lipid metabolism [6, 69, 72], underscoring the importance of TDP-43 in adult development.

With advancing age, TDP-43 is correlated with certain diseases (Fig. 4F), including neurodegenerative disorders and tumors, often accompanied by abnormal changes in its expression or localization. TDP-43 is a highly conserved gene with strict and precise gene expression regulation mechanisms, and even heterozygous knockout of TDP-43 does not affect its expression. This suggests that the self-regulatory capacity of TDP-43 may diminish with age, and disturbances in TDP-43 protein homeostasis can contribute to the development of various diseases, potentially leading to death. Overall, considering its extensive involvement in different life stages, it is plausible to consider that TDP-43 is a key regulatory gene involved in the entire process of mammalian life, from the very beginning to the very end. Future research should focus on the functions of TDP-43 in other contexts, especially in response to stressful conditions.

### Implications from TDP-43 study

The above studies emphasize the diverse and sometimes opposing functions of TDP-43 in various tissues and developmental stages, which may be attributed to the following factors:

1. TDP-43 exhibits stage-specific functions. In degenerative diseases, TDP-43 dysfunction typically occurs in middle to old age. Studies have indicated that the absence of TDP-43 can lead to age-related neuronal degeneration [112]. Comparisons among different-aged patients with ALS have revealed that TDP-43 pathology is more severe in elderly ALS patients. TDP-43 deficient mice also exhibit progressive motor dysfunction and neuropathological changes. Similarly, the consequences of TDP-43 deficiency in oligodendrocytes depend on their maturation stage. Early deletion leads to progressive degeneration of mature oligodendrocytes, leading to seizure and premature death. In contrast, late deletions retain oligodendrocytes to a large extent, and mice survive without seizure, indicating that TDP-43 may be dispensable in mature oligodendrocytes [113].
2. The function of TDP-43 is context-dependent and influenced by basal expression levels in cells and tissues. Notably, in nerve cells, genome RNA cross-linking immunoprecipitation (HITS-CLIP) technology has revealed that TDP-43 can bind with more than 6 000 mRNA targets, representing about 30% of the entire transcriptome [114, 115], thus suggesting the importance of TDP-43 in the nervous system. Regarding breast lactation, TDP-43 can stabilize the mRNA expression levels of *Btn1a1* and *Xdh*, thereby maintaining normal initiation of lactation. Notably, these two genes are transcriptionally activated exclusively in breast lactation cells during lactation, illustrating that TDP-43 regulates their expression through post-transcriptional mechanisms [8]. Furthermore, the cellular expression levels of genes can vary among different cell types, resulting in distinct downstream targets regulated by TDP-43 and consequently exhibiting diverse functions.
3. TDP-43 serves a critical function in the cellular stress response. Notably, the TDP-43 protein can sense intracellular signals, such as misfolding proteins Sup35, Pab1, and Pub1 [116]. In ovariectomized mice, progesterone treatment can promote TDP-43 expression, suggesting that TDP-43 may be regulated by progesterone [117]. TDP-43 can also sense extracellular signals, such as viral infections [2, 93, 95] and oxygen free radicals [118]. Moreover, TDP-43 is defined as a component of stress granules [119, 120] and participates in cellular liquid-liquid phase separation [121, 122]. Under a certain degree of internal and external stimuli, TDP-43 maintains a relatively stable expression state through self-regulation of mRNA, enabling normal responses to relevant stimuli and maintenance of life processes. However, when cells are subjected to intense stimulation or long-term imbalance, the expression or subcellular localization of TDP-43 may be disrupted, leading to disease manifestation. Neural cells continuously receive internal signals from synapses, and TDP 43 serves as a stress sensor gene, detecting and responding to specific stimuli at precise times and in specific cell types. This stress response mechanism is vital for maintaining a steady state during cellular processes.

## Data Availability

Not applicable.
